# Scale of perception of family self-efficacy in home care for children on peritoneal dialysis

**DOI:** 10.1590/0034-7167-2023-0329

**Published:** 2025-03-10

**Authors:** Lígia Simões Ferreira, Myriam Aparecida Mandetta, Maria Magda Ferreira Gomes Balieiro, Francisneide Gomes Pego do Nascimento, Maria Angélica Marcheti

**Affiliations:** I Universidade Federal de Mato Grosso do Sul. Campo Grande, Mato Grosso do Sul, Brazil; II Universidade Federal de São Paulo. São Paulo, São Paulo, Brazil

**Keywords:** Child, Peritoneal Dialysis, Pediatric Nursing, Renal Insufficiency, Chronic, Validation Study, Niño, Diálisis Peritoneal, Enfermería Pediátrica, Insuficiencia Renal Crónica, Estudio de Validación

## Abstract

**Objectives:**

to validate an instrument for family self-efficacy perception in home care for children on peritoneal dialysis.

**Methods:**

a methodological study. The theoretical framework was presented, conducted through a literature review, field research, item development, content validity by a committee of judges and semantic analysis with families. The inter-judge agreement percentage was established at 80%, the Content Validity Index at 0.8 and the Kappa Index at 1.0.

**Results:**

the scale items were developed based on the definition of the family self-efficacy construct, covering four dimensions that correspond to the care behaviors of family members. Content validity obtained 84% agreement among judges, with a Content Validity Index of 0.84 and a total Kappa of 0.70. In semantic analysis, 100% agreement was found.

**Conclusions:**

the instrument showed good agreement among judges and the target population.

## RESUMEN

**Objetivos:**

validar el instrumento de percepción de autoeficacia familiar en el cuidado domiciliario de niños en diálisis peritoneal.

**Métodos:**

estudio metodológico. Se presentó el polo teórico, realizado a través de revisión de literatura, investigación de campo, elaboración de ítems, validación de contenido por un comité de jueces y análisis semántico con familias. El porcentaje de acuerdo entre jueces se estableció en 80%, el Índice de Validez de Contenido en 0,8 y el Índice Kappa en 1,0.

**Resultados:**

los ítems de la escala fueron creados a partir de la definición del constructo de autoeficacia familiar, abarcando cuatro dimensiones que corresponden a las conductas de cuidado de los miembros de la familia. La validación de contenido logró un 84% de acuerdo entre jueces, con un Índice de Validez de Contenido de 0,84 y un Kappa total de 0,70. En el análisis semántico hubo 100% de acuerdo.

**Conclusiones:**

el instrumento mostró buena concordancia entre los jueces y la población objetivo.

## INTRODUCTION

In Brazil, the experience of parents caring for a child with Chronic Kidney Disease (CKD) on peritoneal dialysis (PD) at home reveals a complex dynamic, due to the overload of demands placed on families, which have to deal with repeated hospitalizations, medication administration at home and rigorous execution of the dialysis procedure. As a consequence, parents may lose their working hours, compromising the family income and imposing challenges on the family. Care demands require family involvement, availability and skills to carry out the treatment appropriately^(1,2)^.

Family participation and involvement are essential for carrying out PD at home, considering that it is a procedure that requires the trust and autonomy of everyone involved^(3,4)^. In this regard, families must receive training, with theoretical and practical activities, that will make them able to continue children’s treatment at home. Thus, at the end, nurses assess the skills acquired by families and release them to continue treatment at home^(2)^.

However, in our experience with patients on automated peritoneal dialysis (APD), in children’s first outpatient visits, families’ narratives reveal the difficulties they face with equipment and devices, as well as reports of doubts, fears and anxiety regarding child health maintenance, complaining about having to learn and adapt to a new routine in a short period of time. To be able to take care of children, families need to develop their confidence and ability to carry it out while maintaining their self-esteem and mental health.

The self-efficacy^(5)^ theoretical framework defines it as “the belief that people have about their ability to perform something that has been assigned to them even in the face of adversity”. Self-efficacy directly affects behavior, as people with higher self-efficacy scores are more likely to persist in difficult tasks.

This concept was applied in the field of family relationships, suggesting that, in order to care for children, parents need to feel capable of carrying out tasks successfully^(6)^. It is essential that nurses recognize situations in which families demonstrate difficulty or lack of knowledge of any stage of the therapeutic process and identify situations in which they need support, information or more time to adapt to the development of specific skills required by APD treatment at home and to feel capable of providing care for children.

The question is how families perceive their self-efficacy to care for children with APD at home, in order to guide nurses’ practice with this population. The theoretical approximation of Bandura’s concept of collective efficacy^(7)^ and the concept of family, from the interactionist perspective, proposed by Angelo^(8)^, allows defining families’ self-efficacy as “the sharing of collaborative actions and caring behaviors that families perceive themselves capable of carrying out, in an alignment of the perspectives of their members, generating a dominant pattern of families”. This definition makes it possible to understand families’ perception of self-efficacy in the situation of having a child with APD at home.

In the nursing discipline, more specifically in the field of family nursing, there has been a growing search for instruments to measure subjective phenomena, in order to guide professional practice^(9,10)^. However, we did not find any instrument to measure families’ perception of self-efficacy in managing children on dialysis treatment at home, which motivated us to develop an instrument based on a literature review and field study.

## OBJECTIVES

To develop and validate an instrument to assess families’ self-efficacy in providing home care to children with PD.

## METHODS

### Ethical aspects

All ethical precepts established in Resolution 510/16 were complied with. The study was conducted in accordance with national and international ethical guidelines and was approved by the *Universidade Federal do Mato Grosso do Sul* Research Ethics Committee.

### Study design

This is a methodological study for validating a measuring instrument. The process of constructing the instrument was based on Bandura’s theoretical-methodological framework of self-efficacy^(5)^ and on the model proposed by Pasquali^(11)^, which advocates the implementation of theoretical, empirical and analytical poles.

This article presents the theoretical framework, conducted through a literature review, field study, item development and content validity with semantic analysis.

### Literature review

A scoping review was carried out guided by the JBI methodology^(12,13)^, which recommends the following stages: problem identification; guiding question elaboration; establishment of descriptors and their synonyms; decision on inclusion and exclusion criteria; and definition of databases to be used and the search strategies. The recommendation guide used for constructing the adopted review was the Preferred Reporting Items for Systematic reviews and Meta-Analyses extension for Scoping Reviews (PRISMA-ScR) – JBI^(14)^.

The problem of this study refers to the way families perceive the challenges, difficulties and barriers to caring for children with APD at home. The guiding question was formulated according to the PCC strategy (P - population: families of children/adolescents undergoing PD; C - concept: difficulties and/or challenges of families in managing PD care; C - context: at home): what are the difficulties and challenges evidenced by families when caring for children/adolescents on PD treatment at home?

The *Biblioteca Digital Brasileira de Teses e Dissertações* (BDTD), Cumulative Index to Nursing and Allied Health Literature (CINAHL), National Library of Medicine/National Institute of Health (PubMed/MEDLINE), Scopus (Elsevier) and Scientific Electronic Library Online (SciELO) databases were used. The search was performed using the following MeSH (Medical Subject Headings) descriptors: parents, family, extended families, families relations, child, children, adolescent, adolescents, teens, dialysis peritoneal, peritoneal dialyses, and difficult, which is an uncontrolled descriptor, using the Boolean operators AND and OR.

Full-text studies, available in full in the databases, with quantitative and qualitative research designs, which addressed the challenges and difficulties of families in relation to child/adolescent care on PD, were included. Review articles, letters to the editor, reviews, books, book chapters and commentaries were excluded.

Data collection was carried out using an instrument developed by the authors, consisting of study title, year, indexed database, authors, country, language, year of publication, sample size and characteristics, methodological approach, objectives, results and conclusions.

Afterwards, the texts were read in full. Those that answered the guiding question established in the protocol were transcribed, coded and subcategorized according to similarities and differences, through qualitative content analysis^(15)^. The webQDA software^(16)^ was used to organize the data.

### Field study

A qualitative study was conducted to understand the experience of families in caring for children on APD.

Participants included families of children with CKD enrolled in the APD program, treated at the pediatric nephrology outpatient clinic of a university hospital of a federal higher education institution in Campo Grande, Mato Grosso do Sul. Families of children on APD at home for at least three months, and children without stable clinical conditions during the data collection period were included.

Families were selected using the purposive sampling technique. They determined who their members were and who would participate, since in this study we consider families as a set of individual subsystems, which are not necessarily blood relatives, but rather with emotional ties, chosen by their own members^(17)^.

Data collection was carried out through semi-structured interviews conducted by two researchers who had no previous relationship with participants. The Informed Consent Form (ICF) was obtained from all individuals involved in the study in writing (signature). Initially, a form containing family and child sociodemographic data was filled out, and then a genogram and an ecomap were jointly constructed. Subsequently, the narrative of families’ experience was explored through the guiding question as follows: what difficulties and/or challenges do you believe compromise or interfere with your families’ ability to care for (child’s name) on PD at home? Other questions were asked in order to broaden understanding from families’ perspective.

Each interview was subsequently transcribed in full by one of the researchers. The readings and notes were taken separately by three researchers and organized at webQDA software. In accordance with the principles of inductive qualitative content analysis, the data were organized into four cognitive processes: comprehension; synthesis; theorization; and recontextualization. In comprehension, the transcribed speeches gave rise to codes representing the central idea; in synthesis, the meanings that emerged were described; in theorization, the data were gathered by similarity, constituting thematic groups; and in recontextualization, it was possible to understand the difficulties and challenges faced by families when caring for a child with APD at home^(15)^.

### Scale item construction

Having carried out literature review and field study, it was possible to propose the definition of the construct, which served as the basis for version I of the scale.

### Content validity

A committee of expert judges was formed, whose participants were nurses with experience in child care in pediatric nephrology, with knowledge about scale construction and family-centered care. The selection criteria were adapted from Fehring^(18)^.

After identifying judges, an individual invitation letter was sent by email. If accepted, the ICF and the instrument for assessing the scale content were forwarded.

The Delphi technique was used to collect data to obtain consensus among judges^(18)^. For data analysis, the Content Validity Index (CVI) and the Kappa Index were applied. The percentage of agreement was set at 80%, the CVI at 0.8 and the K Index in the range of 0.61 to 1.0.

### Semantic analysis

Families of children on PD at home participated. Family members of children enrolled in the PD program at home for more than three months, family members of children who had undergone transplants less than six months ago, and family members over 18 years of age or who had been emancipated were included. Families of children diagnosed as beyond the possibility of cure and/or with health complications, admitted to an intensive care unit were excluded.

For data collection, the brainstorming technique^(11)^ was used, which guides a meeting with the lowest stratum (in terms of abilities) of the target population first and, later, with the highest stratum (in terms of abilities) of this same population, to verify whether the items are understandable for all involved.

After suggestions from family members and agreement among judges, the content validity process was concluded.

## RESULTS

### Literature review

**Figure 1:**
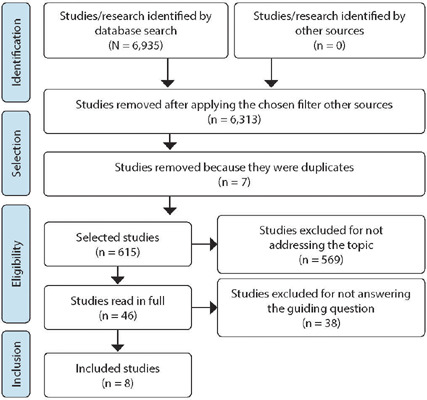
Flowchart of the process of identification, selection and inclusion of studies, prepared based on the Preferred Reporting Items for Systematic reviews and Meta-Analyses extension for Scoping Reviews, Campo Grande, Mato Grosso do Sul, Brazil, 2019

A total of 6,935 studies were retrieved. Of these, 6,313 did not meet pre-established criteria. After discarding ineligible studies, 622 remained. Seven studies were excluded because they were duplicates, and 569 studies whose titles did not correspond to the study theme. Thus, 46 studies were eligible for full reading. Of these, 38 did not answer the guiding question. In the end, eight articles were included, six articles and two full dissertations (Figure 1).

Among the eight studies analyzed, nursing was the field that presented the largest number of studies on the subject (50%), followed by medicine, with 37.5%, and psychology, with 12.5%. Qualitative studies prevailed, being represented by 87.5%, whereas quantitative studies were represented by 12.5%.

From the analysis carried out, four analytical categories emerged:

1. Compromise of families’ social relationships, in which they suffer from restrictions on leisure activities, loss of freedom and social contacts.
2. Compromise in family dynamics, imposing an overload of demands on families, especially on primary caregivers, who become physically and emotionally exhausted due to the need to fulfill an inflexible and complex daily routine of care that demands full dedication to children, leaving the other family members in the background. Routine of families becomes impaired and there is a compromise in work activities, generating a financial imbalance for families. Changes in routine of children with CKD caused by the disease and treatment and changes in the home to adapt to the environment impose more burden on families, as do differences of opinion and marital conflicts generated by changes in family life.
3. Feelings experienced with the PD experience at home manifested by fear of handling equipment at home; fear of being abandoned by the team and fear of infection; hopelessness and fear of a child’s death due to the delay in transplant.
4. Lack of support from the team due to little support from the network and imposition of child care by the team, perceived by families as one of the difficulties in learning and being able to take on the responsibility of caring for children at home.

### Field study

Participants were two mothers, two fathers and one grandmother, aged between 28 and 50 years old, i.e., five members of families with three children assisted by the APD program at home, aged 4, 5 and 11 years old. As for the level of education of family representatives, two had not completed elementary school, one had completed elementary school, one had completed high school and one had completed higher education. The interviews lasted an average of 50 minutes.

Of these children, one was diagnosed with CKD at 3 years and 8 months of undetermined cause, one at 1 year and 2 months, and the other at 8 years of age, all caused by glomerulopathy. Two families reported having jobs without a fixed income, and the other, depending on government aid. All of them rely on financial support from other members of their extended families when they need it.

From field study, it was possible to deepen the knowledge about families’ experiences in this context. Analytical categories represent the difficulties and challenges faced by families in caring for children on PD at home, family functioning and challenging situations that arise due to changes in children’s health condition, the need to adapt family routines to manage dialysis treatment, the use of intra and extra-family resources to deal with the new situation and the relationship with the healthcare team (Chart 1).

**Chart 1:** Analytical categories, Campo Grande, Mato Grosso do Sul, Brazil, 2020

Subcategories	Categories	Families’ speeches
Lack of planning for family routine Re-admissions to hospital Having to care for other family members Loss of children’s childhood Sadness due to the loss of family leisure Need to set limits for the child Different opinions among family members Harmed marital relationship Social isolation Difficulty accepting children’s illness Re-signification of the experience	Challenging situations in family dynamics and functioning	*Her hospitalizations are definitely the biggest test of fire, especially because everything comes to a standstill, I can’t work, her mother stays in the hospital with her.* (Father - Family 1) *We’ve tried going out together. He loves fishing, we’ve thought about going and leaving her with my mother, but I can’t. When I go to the city to buy something, I already want to turn back halfway.* (Mother - Family 1) *Believe that whatever is happening will pass, have that positivity inside you, it will get better.* (Father - Family 1)
Fear of machine failure Fear of power outages in the home Having to perform the dialysis procedure at home without help Physical fatigue due to treatment demands Fear of infection Fear that the catheter will stop working Modification of the home environment to care for children Control of children’s dietary restrictions Control of children’s fluid intake Concern about children’s future Faith to help in coping with the situation	Treatment adaptation and management	*When there is a little hose that is bent, it is tight. I am afraid that something out of the ordinary will happen and I am not there.* (Father - Family 2) *The biggest problem today, besides the fear of an infection, is a problem with her catheter* [clogging]. (Mother - Family 3) *Her mother was going to put her in her room, and she said, “Oh, Mom, I will be alone in the room* [...] *we had to adapt a room in the living room to stay with her* [...] *she adapted grandma’s living room.* (Mother - Family 1) *I think the worst thing we went through was having to rid my daughter of* [denying her] *water. It’s not ice cream, it’s water, it’s something that hurts a lot, telling my daughter, no, you can’t drink water. Oh my God*! (Grandmother - Family 1)
Support from the public network Unable to work Financial shortages Specialized care outside the home	Intra and extra-family resources	*Most of the time I have to buy expensive medicines* [...] *I’m trying to retire her, which isn’t easy* [...] *my mother is always with me and helps me financially when I need it.* (Father - Family 2) *We buy what the health center doesn’t provide, so we held a bingo for her to raise money to buy her medications.* (Father - Family 2)
Discrepancies in information No help from the team to resolve doubts Demanding team	Relationship with the healthcare team	*The intern said, “She needs to drink a lot of water* [...] *pee a lot to release bad things”* [...] *I asked, “Doctor. Is there something wrong, because if I’m giving her the right medications, I’m not letting her run out of medications, I’m following her diet correctly, I’m doing everything right with her dialysis, why is she in this state?”.* (Mother - Family 1) *I feel taken care of, whenever I need them, they’ll take care of me, she’s already told me, at any time. There was one time when the little girl was in pain, I called after two in the morning, she* [the nurse] *woke up and gave me instructions.* (Mother - Family 3)

### Scale item elaboration

The synthesis of the analytical categories identified in the literature review and in the field research allowed the theoretical definition of the construct of self-efficacy of families in the care of children with CKD on PD at home.

This construct is defined as the care behaviors exhibited by family members of a child with kidney disease in order to care for the child at home. The alignment of family members’ perspectives constitutes their perception of self-efficacy in the face of challenging situations, including those pertaining to their dynamics and functioning, as well as in the adaptation and management of the child’s treatment, in obtaining intra- and extra-family resources, and in their relationship with the health care team.

With this definition, we proceeded to develop items that would allow us to measure this collective phenomenon of families. Version I of the self-efficacy perception scale was composed of 27 items, which correspond to the caring behaviors of family members perceived in the experience. The items were initially subdivided into four dimensions: Difficulties that interfere with family dynamics and functioning; Challenges experienced in adapting and managing treatment; Difficulty in maintaining intra and extra-family resources; Challenges in the relationship with the healthcare team (Quadro 2).

**Chart 2:** Distribution of items in version I and their respective dimensions, Campo Grande, Mato Grosso do Sul, Brazil, 2020

Items	Dimension
I have a different opinion than other members of my family about child care. I feel overwhelmed with household chores. I have to set limits for children. I have other children to care for. I feel overwhelmed with my work. I feel sad because I cannot take my child for walks, to play, to travel and to swim. I cannot change my dialysis routine to go out with my child. I feel isolated from my friends because of caring for my child. I am frequently readmitted to hospital.	Difficulties that interfere with family dynamics and functioning
I have no one to share the care of my child with. I have to perform the dialysis procedure at home without help from another person. I have to change the home environment to care for children. The machine fails. Children show some sign of infection (fever, stomach pain, vomiting, body pain, pus in the catheter). I feel tired when turning the dialysis machine on and off. There is a power outage in the house. I feel responsible for peritoneal catheter contamination. I have to control the amount of fluid my child is given. I am afraid the catheter will stop working. I fear for my child’s future (dialysis, transplant, etc.). Transplant is taking a long time to happen.	Challenges experienced in adapting and managing treatment
I am worried about having to wait for medications in the public health system. I don’t have enough money to cover my family’s daily needs. I have to wait for medications in the public health system (with no guarantee of getting them).	Difficulty in maintaining intra and extra-family resources
The team imposes rules without talking to me first. I do not have professional help to answer my questions during peritoneal dialysis. The information and guidance from professionals regarding child care is different.	Challenges in the relationship with the healthcare team

### Content validity

Five experts/judges participated, all of whom were nurses with master’s degrees and professional experience in child health. Four had experience in scale construction and family-centered care, and one had experience in pediatric nephrology. Two had a doctoral degree, one had a post-doctoral degree and one had the highest teaching degree in Brazil (*livre docência*).

The Delphi technique was used as a methodological tool, carried out in three rounds in search of consensus among expert/judge opinions. In the first round, only items 12 and 22 reached a CVI of 0.80, whereas the others varied between 0.20 and 0.60. The total CVI of the scale was 0.42. Given these results, the researchers compiled and qualitatively analyzed the elements that did not reach 80% agreement (CVI of 0.8). The suggestions were grouped and readjusted, with item 10 being added to item 14 and item 25 to item 15. No items were excluded. Item 22, despite having reached 80% agreement, was sent again for analysis, since all items were changed to the first person plural, with the exception of item 12, which was kept unchanged. In the second round, the presentation statement failed to reach the minimum percentage of agreement, maintaining CVI = 0.60. As for the 24 items, five presented perfect agreement (CVI = 1); 14 reached 80% agreement among judges; three obtained CVI = 0.60; and for items 05 and 23, changes were indicated by unanimous decision of judges. In the third round, the presentation statement and the items that had agreement below 80% and CVI less than 0.8 managed to reach the established.

Once this stage was completed, the Kappa Index was calculated. Items 03, 04, 11, 12, 14, 15, 18, 19, 22 and 27 were written in a way that would be more consistent with the theoretical framework used and to be better understood by families.

Among the 28 items of the instrument, nine presented a perfect level of agreement, and 19 presented a fair level of agreement. However, when the total CVI of the scale was calculated, the value was 0.86 and the total Kappa Index was 0.72, demonstrating that version III obtained good agreement among judges (Table 1).

**Table 1:** Percentage of agreement, Validity Confidence Index and Kappa of items of version III, Campo Grande, Mato Grosso do Sul, Brazil, 2020

Items	Agreement %	CVI	Kappa
On a daily basis, your family needs to care for a child with kidney disease, including performing peritoneal dialysis at home. We would like to know how confident you feel about performing the procedures and caring for this child at home, in each of the situations described below.			
Please read each situation carefully and mark with an X the answer that you consider most appropriate. There are no right or wrong answers. What is important for us is to know how you feel, what you think and how you act when caring for a child. Please read each situation carefully and mark with an X the answer that you consider most appropriate. It is important to remember that: 0 (zero) means no confidence and 100 (one hundred), total confidence.	100	1.00	1.00
How confident does your family have that you can continue to care for (child’s name) at home when:	100	1.00	1.00
1. We do not have other people to help us with child care (feeding, hygiene, rest, etc.)	80	0.80	0.60
2. We have to perform child dialysis at home, without help from anyone else.	80	0.80	0.60
3. We have to deal with failures that occur in the dialysis machine.	80	0.80	0.60
4. The team determines the rules (time to start dialysis, dressing technique) without talking to us first.	80	0.80	0.60
5. Children show any signs of infection (fever, abdominal pain, vomiting, diarrhea, body pain, pus in the catheter, drainage of darker fluid).	80	0.80	0.60
6. We do not have professional help to answer our questions during home dialysis.	80	0.80	0.60
7. We have different opinions in the family about child care.	100	1.00	1.00
8. We are tired of having to turn the dialysis machine on and off every day.	100	1.00	1.00
9. We have to change the environment in the house to provide care for children on dialysis.	100	1.00	1.00
10. We have to get the children’s attention, even though we know that they have different needs than other children their age.	80	0.80	0.60
11. There is a power outage in the house.	100	1.00	1.00
12. We have other children who need our care and guidance, which increases our responsibility and fatigue.	80	0.80	0.60
13. We feel overwhelmed by having to care for children on dialysis, do household chores and have to work outside the home.	80	0.80	0.60
14. We worry about having to wait for medication from the public health system, without any guarantee of getting it.	80	0.80	0.60
15. We worry about not having enough money to meet the daily needs of families and sick children.	80	0.80	0.60
16. We feel sad because dialysis limits the leisure activities of our family and the child.	80	0.80	0.60
17. We are unable to change the dialysis routine so that we can all go out together with children.	80	0.80	0.60
18. We feel isolated from friends and family because of child care.	80	0.80	0.60
19. We notice differences in the guidance given by health professionals about child care and we do not know which guidance to follow.	80	0.80	0.60
20. We feel guilty when the peritoneal catheter becomes contaminated.	80	0.80	0.60
21. We are afraid that the catheter will stop working.	100	1.00	1.00
22. We have to control the amount of liquid children can drink.	100	1.00	1.00
23. We receive news that a child needs to be readmitted to the hospital.	80	0.80	0.60
24. We are afraid of having to change from home dialysis to hemodialysis.	80	0.80	0.60
25. We are afraid of the transplant surgery.	100	1.00	1.00
26. We are worried about the delay in performing transplant.	80	0.80	0.60
Total		0.86	0.72

### Semantic analysis

Three families participated in semantic analysis, totaling five members. Of these, four were members of families of children on APD at home for more than six months, and one was a member of the family of a child who had remained on continuous outpatient PD for one year, followed by four years on APD. Afterward, children underwent a kidney transplant during the execution of this study, remaining off treatment for six months.

In the first round, changes were suggested to the fourth item, “The team imposes rules without talking to me”, to “The team forces me to follow the rules, without me being able to give my opinion”. Furthermore, there were also suggestions for changes to item 6 to change the words “at the time of peritoneal dialysis” to “at the time of dialysis”. Item 9, “I have to change the environment”, was changed to “I have to change the environment”. Item 13, “I have other children to care for”, was changed to “I understand that my work outside the home, housework and child care make me feel overwhelmed”. And for item 17, the text “I feel sad because I cannot take my child for walks, to play, to travel and to swim” was modified to “I feel sad because the child is deprived of some activities that are common for them, such as swimming, which is prohibited”. Item 20 was reworded for better understanding; therefore, the text “the information and guidance from professionals regarding child care are different” was modified to “we noticed differences in the guidance on child care given by health professionals and we do not know which guidance to follow”. Finally, there was a request to modify the word “fear” in item 26, as it is difficult to understand, and it was changed to “we are concerned about the delay in carrying out the transplant”. In the second round, 100% of participants agreed on all items. The families did not report any difficulty in understanding or interpreting the gradation of the responses.

In this way, version III of the instrument was validated by experts and the target population.

## DISCUSSION

The study allowed a theoretical definition of the construct of self-efficacy of families in the care of children with CKD on PD at home, enabling elaborating the scale items, validated by a committee of judges with an agreement above 80%.

The rigor for the methodological construction was fundamental and challenging, because, according to Pasquali, there are three difficult moments in the process of developing an instrument: the empirical collection of information to transform the construct into items; the definition of the construct clearly; and statistical analysis to prove whether these items represent the construct^(11)^.

The development of an instrument to identify families’ perceptions of the situation is essential, as it recognizes families as partners of the healthcare team in promoting the best care for children; it respects families’ time and prepares them with dignity so that they feel capable of taking on child care at home, based on sharing of information and acquisition of knowledge, which empowers them to make decisions about child care, in line with patient and family-centered care model recommendations. This model is recognized as one of the best practices to be applied in different care contexts^(19)^.

It is known that the experience of care in this context is complex for families, who need to be well prepared to take on the challenges of daily child care. Thus, the scale presents what family members experience in their experience with a child with CKD undergoing continuous treatment, revealing a strong tension, which can compromise their self-efficacy, resulting from difficulties that interfere with family dynamics and functioning, the challenges experienced in adapting and managing the children under treatment, the difficulty in maintaining intra and extra family resources, and the challenges in the relationship with the healthcare team.

Studies^(20,21)^ have sought to identify the sources of distress experienced by parents of children with chronic dialysis, such as illness-related distress, personal struggles, family structure, lack of resources, and unrealistic social expectations. The authors emphasize the importance of nurses recognizing the experience of families and implementing practices that foster more effective communication and better psycho-emotional support for families.

Therefore, for nurses working in pediatric nephrology, having a validated instrument that assesses families’ readiness to manage children at home is a fundamental resource.

The proposal of an instrument to measure families’ perception of their ability to care for children in the face of the difficulties imposed by disease diagnosis and treatment adds value to nursing science, becoming useful in helping nurses to identify families’ strengths and vulnerabilities and to plan cooperative and aligned actions among their members to modify treatment trajectory and outcome.

However, it is not enough to advocate for patient and family participation in healthcare decision-making; it is necessary to ensure that they have the knowledge necessary for genuine engagement. Healthcare professionals need to move forward with a new emerging issue, which is the importance of ensuring health literacy among patients and families^(22)^. This concept, defined by the World Health Organization^(23)^ as “the ability of individuals to access, process, and use health information and services to make health-related decisions”, is essential, as it supports the patient and family-centered care assumptions, giving the family a voice to process information and learn how to manage child care.

When we bring these issues to families of children in the context of CKD, it becomes clear that, in order to acquire self-efficacy for care, there must be a change in behavior that allows the acquisition of the ability to exercise control over how they function and behave, as well as over the events in their lives.

### Study limitations

A limitation of this study refers to the process of constructing the scale, which involved families representing a single Brazilian state, making it necessary to expand to other regions of Brazil, in order to include elements of the diverse culture of our country.

### Contributions to nursing, health or public policy

The validated instrument can contribute to assessing families’ perception of their ability to manage the care of children on PD, assisting healthcare professionals in proposing actions that favor the self-efficacy of families in caring for children and adolescents undergoing PD at home.

## CONCLUSIONS

The Scale of Perception of Family Self-Efficacy in Home Care for Children on Peritoneal Dialysis (PAFam-CDR-DP) was developed for the purpose of measuring the perceived capability of families in caring for children with PD at home. Content validity and semantic analysis indicated that the scale is appropriate and understandable to families experiencing this context, and encompasses the difficulties, challenges and impediments that need to be overcome by them.

It is recommended to continue the study fulfilling the analytical pole, aiming to carry out psychometric tests for using the scale in clinical practice of nurses with families in this context.
