# Supplementary material for: Scale of perception of family self-efficacy in home care for children on peritoneal dialysis
Source: Rev Bras Enferm. 2025 Mar 10;78(1):e20230329. doi: 10.1590/0034-7167-2023-0329 (PMC11893060; doi:10.1590/0034-7167-2023-0329)
Supplement: Translation

# Translation: Escala de percepção da autoeficácia da família no cuidado domiciliar à criança em diálise peritoneal

**DOI:** 10.1590/0034-7167-2023-0329pt

**Authors:** Lígia Simões Ferreira, Myriam Aparecida Mandetta, Maria Magda Ferreira Gomes Balieiro, Francisneide Gomes Pego do Nascimento, Maria Angélica Marcheti

**Affiliations:** I Universidade Federal de Mato Grosso do Sul. Campo Grande, Mato Grosso do Sul, Brasil; II Universidade Federal de São Paulo. São Paulo, São Paulo, Brasil

**Keywords:** Criança, Diálise Peritoneal, Enfermagem Pediátrica, Doença Renal Crônica, Estudo de Validação

## Abstract

**Objetivos:**

validar o instrumento de percepção da autoeficácia da família no cuidado domiciliar à criança em diálise peritoneal.

**Métodos:**

estudo metodológico. Apresentou-se o polo teórico, conduzido por meio de revisão da literatura, pesquisa de campo, elaboração dos itens, validação de conteúdo por um comitê de juízes e análise semântica com famílias. Estabeleceu-se o percentual de concordância inter-juízes de 80%, o Índice de Validade de Conteúdo de 0,8 e o Índice Kappa de 1,0.

**Resultados:**

os itens da escala foram elaborados a partir da definição do construto autoeficácia da família, contemplando quatro dimensões que correspondem aos comportamentos de cuidado dos membros da família. A validação de conteúdo obteve 84% de concordância entre os juízes, com Índice de Validade de Conteúdo de 0,84 e Kappa total de 0,70. Na análise semântica, verificou-se concordância de 100%.

**Conclusões:**

o instrumento apresentou boa concordância entre os juízes e população-alvo.

## INTRODUÇÃO

No Brasil, a experiência dos pais em cuidar da criança com Doença Renal Crônica (DRC) em diálise peritoneal (DP) no domicílio revela uma dinâmica complexa, devido à sobrecarga de demandas impostas sobre a família, que tem de conviver com internações repetidas, administração de medicamentos no domicílio e execução rigorosa do procedimento dialítico. Como consequência, pode haver a perda da jornada de trabalho dos pais, com comprometimento da renda familiar, impondo desafios à família. As demandas de cuidados exigem da família envolvimento, disponibilidade e habilidades para realizar a terapêutica de modo adequado^(1,2)^.

A participação e o envolvimento da família são imprescindíveis para a realização da DP no domicílio, considerando que se trata de procedimento que exige a confiança e autonomia de todos os envolvidos^(3,4)^. Nesse sentido, a família deve receber uma capacitação, com atividades teórico-práticas, que a torna apta para dar continuidade ao tratamento da criança no domicílio. Assim, ao final, o enfermeiro avalia as habilidades adquiridas pela família e a libera para assumir a continuidade do tratamento na residência^(2)^.

Entretanto, em nossa experiência com pacientes em diálise peritoneal automatizada (DPA), nos primeiros retornos ambulatoriais da criança, observam-se nas narrativas das famílias as dificuldades enfrentadas por elas com os equipamentos e dispositivos, assim como relatam dúvidas, temores e ansiedade quanto à manutenção da saúde da criança, queixando-se da obrigatoriedade em ter de aprender e se adaptar à nova rotina em um curto período de tempo. Para dar conta do cuidado da criança, a família precisa desenvolver sua confiança e capacidade para executá-la, mantendo sua autoestima e saúde mental.

O referencial teórico de autoeficácia^(5)^ a define como “a crença que as pessoas têm sobre sua capacidade em desempenhar algo que lhes foi designado mesmo diante de adversidades”. A autoeficácia afeta diretamente o comportamento, pois pessoas com maior escore de autoeficácia têm maior probabilidade de persistir mediante tarefas difíceis.

Tal conceito tem sido aplicado no campo das relações familiares, sugerindo que, para cuidar de crianças, os pais precisam se sentir capazes de realizar tarefas com sucesso^(6)^. Torna-se fundamental que o enfermeiro perceba situações em que a família demonstre dificuldade ou desconhecimento de qualquer etapa do processo terapêutico e identifique situações em que ela necessite de apoio, informação ou de mais tempo para adaptação para o desenvolvimento de habilidades específicas impostas pelo tratamento de DPA no domicílio e para que se perceba capaz para realizar o cuidado da criança.

Questiona-se como a família percebe sua autoeficácia para cuidar da criança em DPA no domicílio, a fim de nortear a prática do enfermeiro com essa população. A aproximação teórica do conceito de eficácia coletiva de Bandura^(7)^ e o conceito de família, na perspectiva interacionista, proposta por Angelo^(8)^, permite a definição de autoeficácia da família como “o compartilhamento de ações colaborativas e os comportamentos de cuidado que a família se percebe capaz de realizar, em um alinhamento das perspectivas de seus membros, gerando um padrão dominante da família”. Tal definição possibilita compreender a percepção da autoeficácia da família na situação de ter uma criança em DPA no domicílio.

Na disciplina de enfermagem, mais especificamente na área da enfermagem da família, tem havido uma crescente busca por instrumentos de medida de fenômenos subjetivos, a fim de nortear a prática dos profissionais^(9,10)^. No entanto, não encontramos nenhum instrumento de medida da percepção da autoeficácia da família no manejo da criança em tratamento dialítico no domicílio, o que nos motivou a construir um instrumento com base em revisão da literatura e estudo de campo.

## OBJETIVOS

Construir e validar um instrumento de percepção da autoeficácia da família no cuidado domiciliar à criança em DP.

## MÉTODOS

### Aspectos éticos

Todos os preceitos éticos estabelecidos na Resolução nº 510/16, foram observados. O estudo foi conduzido de acordo com as diretrizes de ética nacionais e internacionais, e foi aprovado pelo Comitê de Ética em Pesquisa da Universidade Federal do Mato Grosso do Sul.

### Delineamento do estudo

Estudo do tipo metodológico para validação de instrumento de medida. O processo de construção do instrumento foi fundamentado no referencial teórico-metodológico de autoeficácia de Bandura^(5)^ e no modelo proposto por Pasquali^(11)^, que preconiza a realização de polos teóricos, empíricos e analíticos.

Neste artigo, apresenta-se o polo teórico, conduzido por meio de revisão da literatura, estudo de campo, elaboração dos itens e validação de conteúdo com análise semântica.

### Revisão da literatura

Realizou-se revisão de escopo, orientada pela metodologia JBI^(12,13)^, que preconiza as seguintes etapas: identificação do problema; elaboração da questão norteadora; estabelecimento dos descritores e suas sinonímias; decisão sobre os critérios de inclusão e exclusão; e definição das bases de dados a serem utilizadas e as estratégias de busca. O guia de recomendação para construção da revisão adotada foi o *Preferred Reporting Items for Systematic reviews and Meta-Analyses extension for Scoping Reviews* (PRISMA-ScR) – JBI^(14)^.

O problema do estudo refere-se à maneira como a família percebe os desafios, dificuldades e barreiras para cuidar da criança em DPA no domicílio. A pergunta norteadora foi formulada de acordo com a estratégia PCC (P - população: famílias de crianças/adolescentes em DP; C - conceito: dificuldades e/ou desafios da família no manejo dos cuidados na DP; C - contexto: no domicílio): quais são as dificuldades e os desafios evidenciados pelas famílias ao cuidar de crianças/adolescentes em tratamento de DP no domicílio?

Foram utilizadas as bases de dados Biblioteca Digital Brasileira de Teses e Dissertações (BDTD), *Cumulative Index to Nursing and Allied Health Literature* (CINAHL), *National Library of Medicine/ National Institute of Health* (PubMed/MEDLINE), Scopus (*Elsevier*) e *Scientific Electronic Library Online* (SciELO). A busca foi realizada utilizando os cruzamentos dos seguintes descritores MeSH (*Medical Subject Headings*): *parents, family, extended families, families relations, child, children, adolescent, adolescents, teens, dialysis peritoneal, peritoneal dialysis*, e *difficult*, que é um descritor não controlado, utilizando os operadores booleanos *AND* e *OR*.

Foram incluídos estudos completos, disponíveis na íntegra nas bases de dados, com desenhos de pesquisa quantitativa e qualitativa, que abordassem os desafios e as dificuldades das famílias em relação aos cuidados de crianças/adolescentes em DP. Foram excluídos artigos de revisão, cartas ao editor, resenhas, livros, capítulos de livros e comentários.

A coleta de dados se deu por meio de instrumento elaborado pelos autores composto por título do estudo, ano, base de dado indexada, autores, país, idioma, ano de publicação, tamanho e características da amostra, abordagem metodológicas, objetivos, resultados e conclusões.

Após, os textos foram lidos na íntegra. Aqueles que respondiam à pergunta norteadora estabelecida no protocolo foram transcritos, codificados e subcategorizados de acordo com similaridade e divergências, por meio de análise qualitativa de conteúdo^(15)^. Utilizou-se o *software webQDA* ^(16)^ para a organização dos dados.

### Estudo de campo

Foi realizado estudo de abordagem qualitativa para compreender a experiência da família no cuidado da criança em DPA.

Os participantes incluíram famílias de crianças com DRC inseridas no programa de DPA, atendidas em ambulatório de nefrologia pediátrica de hospital universitário de instituição federal de ensino superior em Campo Grande, Mato Grosso do Sul. Foram incluídas famílias de crianças em DPA no domicílio por, no mínimo, três meses, e crianças sem condições clínicas estáveis durante o período da coleta de dados.

As famílias foram selecionadas utilizando a técnica de amostragem intencional. Elas determinaram quem eram os seus membros e quais iriam participar, já que, neste estudo, consideramos a família como um conjunto de subsistemas individuais, que não necessariamente são pessoas consanguíneas, mas sim com vínculos emocionais, escolhidos por seus próprios membros^(17)^.

A coleta de dados foi realizada por meio de entrevista semiestruturada conduzida por duas pesquisadoras que não tiveram relação anterior com os participantes. O Termo de Consentimento Livre e Esclarecido (TCLE) foi obtido de todos os indivíduos envolvidos no estudo por meio escrito (assinatura). Inicialmente, era preenchido um formulário contendo dados sociodemográficos da família e da criança, e posteriormente realizava-se a construção conjunta do genograma e ecomapa. Em seguida, explorava-se a narrativa da experiência da família por meio da questão norteadora: quais dificuldades e/ou desafios vocês acreditam comprometer ou interferir na capacidade da sua família ao cuidar da (nome da criança) em DP no domicílio? Outras questões foram formuladas, a fim de ampliar a compreensão na perspectiva da família.

Posteriormente, cada entrevista foi transcrita na íntegra por uma das pesquisadoras. As leituras e anotações foram realizadas separadamente por três pesquisadoras e organizadas no *software web QDA* ^(16)^.

De acordo com os princípios da análise qualitativa de conteúdo do tipo indutiva, os dados foram organizados em quatro processos cognitivos: compreensão; sintetização; teorização; e recontextualização. Na compreensão, as falas transcritas deram origem a códigos representativos da ideia central; na sintetização, os significados emergidos foram descritos; na teorização, os dados foram reunidos por similaridade, constituindo os grupos temáticos; e na recontextualização, foi possível a compreensão das dificuldades e dos desafios enfrentados pela família ao cuidarem de criança em DPA no domicílio^(15)^.

### Construção dos itens da escala

Tendo realizado a revisão da literatura e o estudo de campo, foi possível propor a definição do construto, que serviu de base para a versão I da escala.

### Validação de conteúdo

Foi composto um comitê de juízes especialistas, cujos participantes foram enfermeiros com experiência em cuidado da criança na área da nefrologia pediátrica, com conhecimento sobre construção de escalas e cuidado centrado na família. Os critérios de escolha foram adaptados de Fehring^(18)^.

Após a identificação dos juízes, enviou-se uma carta convite individual por correio eletrônico. Em caso de aceite, encaminhavam-se o TCLE e o instrumento para avaliação do conteúdo da escala.

Na coleta de dados, para a obtenção de consenso entre os juízes, utilizou-se a técnica Delphi^(18)^. Para a análise dos dados, aplicaram-se o Índice de Validade de Conteúdo (IVC) e o Índice Kappa. Estabeleceram-se a porcentagem de concordância de 80%, o IVC de 0,8 e o Índice Kappa na faixa de 0,61 a 1,0.

### Análise semântica

Participaram famílias de crianças em DP no domicílio. Foram incluídos membros de família de crianças inseridas no programa de DP no domicílio a mais de três meses, membros de família de crianças transplantadas a menos de seis meses e familiares maiores de 18 anos ou emancipados. Foram excluídas famílias de crianças diagnosticadas como fora de possibilidades de cura e/ou com intercorrências de saúde, internadas em unidade de cuidados intensivos.

Para a coleta de dados, utilizou-se a técnica de *brainstorming* ^(11)^, que orienta um encontro com o estrato mais baixo (de habilidades) da população-alvo primeiramente e, posteriormente, com o estrato mais alto (de habilidades) dessa mesma população, para verificar se os itens estão compreensíveis para todos os envolvidos.

Após as sugestões dos membros das famílias e a concordância dos juízes, o processo de validação de conteúdo foi concluído.

## RESULTADOS

### Revisão da literatura

Foram recuperados um total de 6.935 estudos. Desses, 6.313 não atenderam aos critérios pré-estabelecidos. Descartados os inelegíveis, restaram 622. Excluíram-se sete estudos, por estarem duplicados, e 569, cujos títulos não correspondiam ao tema do estudo. Dessa maneira, um total de 46 estudos foram elegíveis para a leitura na íntegra. Desses, 38 não responderam à pergunta norteadora. Ao final, oito artigos foram incluídos, sendo seis artigos e duas dissertações na íntegra (Figura 1).

**Figura 1:** Fluxograma do processo de identificação, seleção e inclusão dos estudos, elaborado a partir da recomendação *Preferred Reporting Items for Systematic reviews and Meta-Analyses extension for Scoping Reviews*, Campo Grande, Mato Grosso do Sul, Brasil, 2019

Entre as oito publicações analisadas, a enfermagem foi a área que apresentou maior número de estudos sobre o tema (50%), seguida da medicina, com 37,5%, e psicologia, com 12,5%. Os estudos de abordagem qualitativa prevaleceram, sendo representados por 87,5%, enquanto que os de abordagem quantitativa foram representados por 12,5%.

Da análise realizada, emergiram quatro categorias analíticas:

1. Comprometimento das relações sociais da família, em que ela sofre pela restrição para atividades de lazer, perda da liberdade e de contatos sociais.
2. Comprometimento na dinâmica familiar, impondo sobrecarga de demandas na família, especialmente ao cuidador principal, que se torna exausto física e emocionalmente em razão da necessidade de cumprir diariamente uma rotina de cuidados inflexível e complexa que exige dedicação integral à criança, deixando os demais membros da família em segundo plano. A rotina da família torna-se prejudicada e há um comprometimento das atividades laborais, gerando um desequilíbrio financeiro da família. As modificações da rotina da criança com DRC causadas pela doença e tratamento e as alterações no domicílio para adequação do ambiente impõem mais sobrecarga à família, assim como as divergências de opiniões e conflitos conjugais gerados pelas mudanças na vida familiar.
3. Sentimentos vivenciados com a experiência de DP no domicílio manifestados por temor no manuseio do equipamento no domicílio; temor de ser abandonada pela equipe e temor da infecção; desesperança e medo da morte do filho pela demora no transplante.
4. Falta de apoio da equipe devido a pouco suporte da rede e imposição de cuidados com a criança pela equipe, percebidos pela família como uma das dificuldades para aprender e ser capaz de dar conta da responsabilidade no cuidado da criança no domicílio.

### Estudo de campo

Participaram duas mães, dois pais e uma avó com idades entre 28 e 50 anos, ou seja, cinco membros de famílias de três crianças atendidas no programa de DPA no domicílio de quatro, cinco e 11 anos de idade. Quanto ao nível de escolaridade dos representantes das famílias, dois haviam cursado o ensino fundamental incompleto, um, o ensino fundamental completo, um, o ensino médio completo, e um, o ensino superior completo. As entrevistas tiveram uma duração média de 50 minutos.

Dessas crianças, uma foi diagnosticada com DRC, aos 3 anos e 8 meses, de causa indeterminada, uma, aos 1 anos e 2 meses, e a outra, com 8 anos, todas causadas por glomerulopatia. Duas famílias relataram ter emprego sem renda fixa, e a outra, depender de auxílios governamentais. Todas contam com o apoio financeiro de outros membros da família ampliada quando necessitam.

A partir do estudo de campo, foi possível aprofundar o conhecimento sobre a experiência da família nesse contexto. As categorias analíticas representam as dificuldades e os desafios enfrentados pela família no cuidado da criança em DP no domicílio, o funcionamento da família e as situações desafiadoras que surgem em virtude das mudanças na condição de saúde da criança, a necessidade de adaptação das rotinas familiares para o manejo do tratamento dialítico, a utilização dos recursos intra e extrafamiliares para lidar com a nova situação e o relacionamento com a equipe de saúde (Quadro 1).

**Quadro 1:** Categorias analíticas, Campo Grande, Mato Grosso do Sul, Brasil, 2020

Subcategorias	Categorias	Vozes da família
Falta de planejamento da rotina familiar Reinternações no hospital Ter de cuidar de outros membros da família Perda da infância do(a) filho(a) Tristeza pela perda de lazer em família Necessidade de dar limites à criança Divergências de opiniões entre os membros da família Relacionamento conjugal prejudicado Isolamento social Dificuldade para aceitar a doença da criança Ressignificação da experiência	Situações desafiadoras na dinâmica e funcionamento familiar	*As internações dela, com certeza, são a maior prova de fogo, até porque paralisa tudo, eu não posso trabalhar, a mãe fica no hospital com ela.* (Pai - Família 1) *A gente já tentou sair juntos. Ele adora pescar, já pensamos em ir e deixar ela com a minha mãe, mas eu não consigo. Quando eu vou na cidade comprar alguma coisa, eu já quero voltar no meio do caminho.* (Mãe - Família 1) *Acreditar que o que está acontecendo vai passar, ter aquela positividade dentro de você, vai ficar melhor*. (Pai - Família 1)
Medo da ocorrência de falha na máquina Temor da falta de energia elétrica na casa Ter de realizar o procedimento dialítico no domicílio sem ajuda Cansaço físico com a demanda do tratamento Pavor da infecção Temor de que o cateter pare de funcionar Modificação do ambiente doméstico para realizar os cuidados com a criança Controle das restrições alimentares da criança Controle da ingestão de líquido da criança Preocupação com o futuro da criança Fé para auxiliar no enfrentamento da situação	Adaptação e manejo do tratamento	*Quando tem alguma mangueirinha que está dobrada, está apertada. Tenho medo de acontecer alguma coisa que esteja fora do normal e eu não estiver lá*. (Pai - Família 2) *O maior problema hoje, além do medo de uma infecção, é um problema no cateter dela* [entupimento]. (Mãe - Família 3) *A mãe ia colocar ela no quarto, e ela falou: “Ah, mãe, lá no quarto eu vou ficar sozinha* [...] *tivemos que adaptar um quarto na sala para ficar com ela* [...] *adaptou a sala da vó.* (Mãe - Família 1) *Acho que a pior coisa que nós passamos foi ter de ridicar* [negar] *água para minha filha. Não é um sorvete, é água, é uma coisa que magoa muito, falar para minha filha, não, você não pode beber água. Minha Nossa Senhora!* (Avó - Família 1)
Apoio da rede pública Sem conseguir trabalhar Escassez financeira Atendimento especializado fora do domicílio	Recursos intra e extrafamiliares	*A maioria das vezes tenho que comprar medicamentos de alto custo* [...] *eu estou tentando aposentar ela, que não é fácil* [...] *a minha mãe está sempre comigo e me ajuda financeiramente quando preciso.* (Pai - Família 2) *A gente compra o que não fornece na casa de saúde, então a gente fez um bingo para ela para poder arrecadar dinheiro para comprar os remédios dela.* (Pai - Família 2)
Divergências de informações Sem ajuda da equipe para tirar dúvidas Equipe exigente	Relacionamento com a equipe de saúde	*A estagiária falou: “Ela tem que beber bastante água* [...] *fazer bastante xixi para liberar coisas ruins”* [...] *eu questionei: “Dr. Tem alguma coisa errada, porque se eu estou dando os remédios certos, não estou deixando faltar os remédios, tô fazendo a dieta dela certinha, tô fazendo a diálise tudo certo, por que ela está nesse ponto?”.* (Mãe - Família 1) *Me sinto atendida, qualquer horário que eu precisar, eles me atendem, ela já falou para mim, a qualquer hora. Teve uma vez que a nenenzinha tava com dor, eu liguei mais de duas horas da manhã, ela* [a enfermeira] *acordou e me orientou.* (Mãe - Família 3)

### Elaboração dos itens da escala

A síntese das categorias analíticas identificadas na revisão da literatura e na pesquisa de campo permitiu a definição teórica do construto da autoeficácia da família no cuidado da criança com doença renal em DP no domicílio.

Este constructo é definido como os comportamentos de cuidado, isto é, a ação cooperativa que se dá entre os membros da família da criança com doença renal para a realização dos cuidados com a criança no domicílio. O alinhamento das perspectivas dos membros da família compõe a percepção da sua autoeficácia diante das situações que os desafiam em sua dinâmica e funcionamento, assim como na adaptação e no manejo do tratamento da criança, na obtenção de recursos intra e extrafamiliares e no relacionamento com a equipe de saúde”.

Com essa definição, prosseguiu-se a elaboração dos itens que permitissem aferir esse fenômeno coletivo da família. A versão I da escala de percepção da autoeficácia foi composta por 27 itens, que correspondem aos comportamentos de cuidado dos membros da família percebidos na experiência. Os itens foram, inicialmente, subdivididos em quatro dimensões: Dificuldades que interferem na dinâmica e funcionamento familiar; Desafios vivenciados na adaptação e manejo do tratamento; Dificuldade para manter os recursos intra e extrafamiliares; Desafios no relacionamento com a equipe de saúde (Quadro 2).

**Quadro 2:** Distribuição dos itens na versão I e sua respectiva dimensão, Campo Grande, Mato Grosso do Sul, Brasil, 2020

Itens	Dimensão
Tenho opinião diferente dos outros membros da minha família sobre os cuidados da criança. Fico sobrecarregado com os afazeres da casa. Tenho de dar limites à criança. Tenho outros filhos para cuidar. Fico sobrecarregado com meu trabalho. Sinto-me triste por não poder levar minha criança para passear, brincar, viajar e nadar. Não consigo modificar a rotina da diálise para sair com a criança. Sinto-me isolada dos amigos por causa dos cuidados com a criança. Reinternações frequentes.	Dificuldades que interferem na dinâmica e funcionamento familiar
Não tenho com quem dividir o cuidado da minha criança. Tenho de realizar o procedimento dialítico no domicílio sem ajuda de outra pessoa. Tenho de modificar o ambiente de casa para realizar os cuidados com a criança. Ocorre falha na máquina. A criança apresenta algum sinal de infecção (febre, dor na barriga, vômito, dor no corpo, pus no cateter). Sinto-me cansado (a) para ligar e desligar a máquina de diálise. Ocorre falta de energia elétrica na casa. Sinto-me responsabilizada pela contaminação do cateter peritoneal. Tenho de controlar a quantidade de líquido para minha criança. Tenho medo do cateter parar de funcionar. Temo pelo futuro da minha criança (diálise, transplante, etc.). O transplante está demorando a acontecer.	Desafios vivenciados na adaptação e manejo do tratamento
Sinto-me preocupada em ter de esperar pelos medicamentos na rede pública. Falta dinheiro para dar conta das necessidades diárias da família. Tenho de esperar pelos medicamentos na rede pública (sem garantia de obtê-los).	Dificuldade para manter os recursos intra e extrafamiliares
A equipe impõe as regras sem conversar comigo antes. Não tenho ajuda profissional para tirar minhas dúvidas na hora da diálise peritoneal. As informações e orientações dos profissionais em relação ao cuidado com a criança são diferentes.	Desafios no relacionamento com a equipe de saúde

### Validação de conteúdo

Participaram cinco especialistas/juízes, sendo todos enfermeiros mestres e com experiência profissional em saúde da criança. Quatro possuíam experiência em construção de escalas e cuidado centrado na família, e um possuía experiência em nefropediatria. Dois possuíam título de doutor, um com pós-doutorado e um com título de livre docente.

A técnica Delphi foi utilizada como ferramenta metodológica, realizada em três rodadas em busca de consenso entre as opiniões dos especialistas/juízes. Na primeira rodada, somente os itens 12 e 22 atingiram o IVC de 0,80, enquanto que os demais variaram entre 0,20 e 0,60. O IVC total da escala foi de 0,42. Diante de tais resultados, as pesquisadoras compilaram e analisaram qualitativamente os elementos que não atingiram a concordância de 80% (IVC de 0,8). As sugestões foram agrupadas e readequadas, ficando o item 10 agregado ao item 14 e o 25 ao 15 item. Nenhum item foi excluído. O item 22, apesar de ter atingido a concordância de 80%, foi encaminhado novamente para análise, pois todos os itens foram passados para a primeira pessoa do plural, com exceção do item 12, que foi mantido sem alteração. Na segunda rodada, o enunciado de apresentação não conseguiu atingir o percentual mínimo de concordância, mantendo IVC = 0,60. Quanto aos 24 itens, cinco apresentaram concordância perfeita (IVC = 1); 14 atingiram 80% de concordância entre os juízes; três obtiveram IVC = 0,60; e para os itens 05 e 23, foram indicadas alterações pela unanimidade dos juízes. Na terceira rodada, o enunciado de apresentação e os itens que estavam com a concordância abaixo de 80% e IVC menor que 0,8 conseguiram atingir o estabelecido.

Com a finalização desse passo, o Índice Kappa foi calculado. Os itens 03, 04, 11, 12, 14, 15, 18, 19, 22 e 27 foram redigidos de forma que ficassem mais condizentes com o referencial teórico utilizado e para ser mais bem compreendido pelas famílias.

Entre os 28 itens do instrumento, nove apresentaram nível de concordância perfeito, e 19 apresentaram uma concordância regular. No entanto, quando se calcula o IVC total da escala, atingem-se o valor de 0,86 e o Kappa total de 0,72, demonstrando que a versão III da escala obteve uma boa concordância entre os juízes (Tabela 1).

**Tabela 1:** Porcentagem de concordância, Índice de Validade de Confiança e Kappa dos itens da versão III, Campo Grande, Mato Grosso do Sul, Brasil, 2020

Itens	Concordância %	IVC	Kappa
No dia a dia, a família de vocês precisa cuidar da criança com doença renal, incluindo também a realização da diálise peritoneal em casa. Gostaríamos de saber o quanto vocês se sentem confiantes para realizar os procedimentos e os cuidados dessa criança em casa, em cada uma das situações descritas abaixo.			
Por favor, leiam com atenção cada situação e marquem com X a resposta que vocês consideram mais adequada. Não há respostas certas ou erradas. O importante para nós é saber como vocês se sentem, o que pensam e como agem ao cuidar da criança. Por favor, leiam com atenção cada situação e marquem com X a resposta que vocês consideram mais adequada. Importante lembrar que: 0 (zero) significa nenhuma confiança e 100 (cem), total confiança.	100	1,00	1,00
Quanta confiança a família de vocês tem de que conseguem continuar a realizar os cuidados da/do (nome da criança) em casa, quando:	100	1,00	1,00
1. Não temos outras pessoas para nos ajudar com os cuidados da criança (alimentação, higiene, repouso, entre outros)	80	0,80	0,60
2. Temos que realizar a diálise da criança em casa, sem ajuda de outra pessoa.	80	0,80	0,60
3. Temos que lidar com as falhas que ocorrem na máquina de diálise.	80	0,80	0,60
4. A equipe determina as regras (horário do início da diálise, técnica do curativo) sem conversar conosco antes.	80	0,80	0,60
5. A criança apresenta algum sinal de infecção (febre, dor abdominal, vômito, diarreia, dor no corpo, pus no cateter, drenagem de líquido mais escuro).	80	0,80	0,60
6. Não temos ajuda profissional para tirar nossas dúvidas durante a diálise em casa.	80	0,80	0,60
7. Temos na família opiniões diferentes sobre os cuidados da criança.	100	1,00	1,00
8. Estamos cansados de ter que ligar e desligar a máquina de diálise diariamente.	100	1,00	1,00
9. Temos que modificar o ambiente da casa para realizar os cuidados da criança em tratamento dialítico.	100	1,00	1,00
10. Temos que chamar a atenção da criança, mesmo sabendo que ela tem necessidades diferentes de outras crianças da sua idade.	80	0,80	0,60
11. Ocorre falta de energia elétrica na casa.	100	1,00	1,00
12. Temos outros filhos que precisam de nossos cuidados e orientações, o que aumenta nossa responsabilidade e cansaço.	80	0,80	0,60
13. Nos sentimos sobrecarregados por ter de cuidar da criança em diálise, dos afazeres da casa e ter que trabalhar fora.	80	0,80	0,60
14. Nos preocupamos em ter que esperar pelos medicamentos na rede pública de saúde, sem a garantia de obtê-los.	80	0,80	0,60
15. Nos preocupamos com a falta de dinheiro para dar conta das necessidades diárias da família e da criança doente.	80	0,80	0,60
16. Sentimos tristeza porque a diálise limita as atividades de lazer da nossa família e da criança.	80	0,80	0,60
17. Não conseguimos modificar a rotina da diálise para sairmos todos juntos com a criança.	80	0,80	0,60
18. Sentimos que ficamos isolados dos amigos e familiares, por causa dos cuidados com a criança.	80	0,80	0,60
19. Percebemos diferenças nas orientações sobre os cuidados da criança dadas pelos profissionais de saúde e ficamos sem saber qual orientação seguir.	80	0,80	0,60
20. Nos sentimos culpados quando ocorre a contaminação do cateter peritoneal.	80	0,80	0,60
21. Temos medo do cateter parar de funcionar.	100	1,00	1,00
22. Temos que controlar a quantidade de líquido que a criança pode tomar.	100	1,00	1,00
23. Recebemos a notícia de que a criança precisa ser reinternada no hospital.	80	0,80	0,60
24. Sentimos medo de ter que mudar da diálise em casa para a hemodiálise.	80	0,80	0,60
25. Sentimos medo da cirurgia do transplante.	100	1,00	1,00
26. Sentimos preocupação com a demora para a realização do transplante.	80	0,80	0,60
Total		0,86	0,72

### Análise semântica

Participaram da análise semântica três famílias, totalizando cinco membros. Desses, quatro eram membros de famílias de criança em DPA no domicílio há mais de seis meses, e um era membro de família de criança que havia permanecido em DP ambulatorial contínua por um ano, seguido de quatro anos em DPA. Após, foi submetida a transplante de rim durante a execução deste estudo, permanecendo seis meses fora de tratamento.

Na primeira rodada, foram sugeridas alterações no quarto item, “A equipe impõe regras sem conversar comigo”, por “A equipe me obriga a cumprir as regras, sem eu poder dar minha opinião”. Ademais, também houve sugestões de alteração no item 6 para trocar as palavras “na hora de diálise peritoneal” por “no momento da diálise”. O item 9, “tenho de modificar o ambiente”, foi alterado para “tenho que modificar o ambiente”. O item 13, “tenho outros filhos para cuidar”, foi alterado para “eu entendo que meu trabalho fora de casa, o trabalho de casa e os cuidados com a criança me fazem ficar sobrecarregada”. E para o item 17, modificou-se o texto “sinto-me triste por não poder levar minha criança para passear, brincar, viajar e nadar” por “sinto-me triste pela criança se privar de algumas atividades comuns para ela, como nadar que é proibido”. O item 20 recebeu nova redação para melhor entendimento; portanto, o texto “as informações e orientações dos profissionais em relação ao cuidado com a criança são diferentes” foi modificado para “percebemos diferenças nas orientações sobre o cuidado da criança dadas pelos profissionais de saúde e ficamos sem saber qual orientação seguir”. Por fim, houve a solicitação para modificar a palavra “temor” do item 26, por ser de difícil compreensão, sendo modificado para “sentimos preocupação com a demora para a realização do transplante”. Na segunda rodada, obteve-se concordância de 100% dos participantes em todos os itens. As famílias não referiram nenhuma dificuldade na compreensão ou na interpretação da gradação das respostas.

Desta maneira, a versão III do instrumento foi validada pelos especialistas e pela população-alvo.

## DISCUSSÃO

O estudo permitiu a definição teórica do construto de autoeficácia da família no cuidado da criança com doença renal em DP no domicílio, possibilitando a elaboração dos itens da escala, validada pelo comitê de juízes com uma concordância acima de 80%.

O rigor para a construção metodológica foi fundamental e desafiador, pois, segundo Pasquali, há três momentos difíceis no processo de elaboração de um instrumento: a coleta empírica de informações para transformar o construto em itens; a definição do construto de forma clara; e a análise estatística, para comprovar se esses itens representam o construto^(11)^.

A construção de um instrumento para identificar a percepção da família sobre a situação torna-se primordial, pois reconhece a família como parceira da equipe de saúde para promover o melhor cuidado à criança; respeita o tempo da família e a prepara com dignidade para que se perceba capaz de assumir o cuidado da criança no domicílio, a partir do compartilhamento de informações e da aquisição de conhecimento, que a torna empoderada para tomar decisões acerca dos cuidados da criança, indo ao encontro do preconizado pelo modelo do cuidado centrado no paciente e família. Este modelo é reconhecido como uma das melhores práticas para ser aplicado nos diferentes contextos de cuidado^(19)^.

Sabe-se que a experiência do cuidado neste contexto é complexa para a família, que precisa estar bem preparada para assumir os desafios no cotidiano do cuidado da criança. Dessa maneira, a escala apresenta o correspondente ao que os membros da família vivenciam em sua experiência com a criança renal crônica em tratamento contínuo, revelando uma forte tensão, que pode comprometer sua autoeficácia, decorrente das dificuldades que interferem na dinâmica e funcionamento familiar, dos desafios vivenciados na adaptação e manejo da criança sob tratamento, da dificuldade para manter os recursos intra e extra familiares, e dos desafios no relacionamento com a equipe de saúde.

Estudos^(20,21)^ realizados buscaram identificar as fontes de *distress* experienciadas por pais de crianças com doença crônica em diálise, tais como angústia relacionada à doença, lutas pessoais, estrutura familiar, falta de recursos e expectativas sociais irrealistas. Os autores reforçam a importância de o enfermeiro reconhecer a experiência da família e realizar uma prática que cultive mais comunicação efetiva e melhor o suporte psicoemocional à família.

Dessa maneira, para os enfermeiros que atuam na área da nefropediatria, ter um instrumento validado que conduza a avaliação da prontidão da família para o manejo da criança no domicílio é um recurso fundamental.

A proposição de um instrumento para medir a percepção da família em relação à sua capacidade para cuidar da criança diante das dificuldades impostas pelo diagnóstico e tratamento da doença agrega valor à ciência da enfermagem, tornando-se útil para ajudar o enfermeiro a identificar as forças e vulnerabilidades da família e a planejar junto com ela ações cooperativas e alinhadas entre seus membros para modificar a trajetória e o desfecho do tratamento.

No entanto, não basta advogar pela participação do paciente e família na tomada de decisão nos cuidados em saúde; é preciso garantir-lhes o conhecimento necessário para que haja um engajamento genuíno. Os profissionais de saúde precisam avançar para uma nova questão emergente, que é a importância da garantia da literacia em saúde dos pacientes e família^(22)^. Este conceito, definido pela Organização Mundial da Saúde^(23)^ como “o grau em que os indivíduos têm a capacidade de obter, processar e entender as informações básicas de saúde para utilizarem os serviços e tomarem decisões adequadas de saúde”, é fundamental, pois corrobora os pressupostos do cuidado centrado no paciente e família, dando voz à família para processar as informações e aprender a manejar o cuidado da criança.

Ao trazermos essas questões para a família da criança no contexto da DRC, torna-se evidente que, para adquirir autoeficácia para o cuidado, é preciso haver mudança de comportamento que permita a aquisição de capacidade para exercer o controle sobre como funciona e se comporta, assim como sobre os eventos de suas vidas.

### Limitações do estudo

Uma limitação deste estudo refere-se ao processo de construção da escala, que envolveu famílias representantes de um único estado brasileiro, sendo necessário ampliar para as demais regiões do Brasil, a fim de incluir elementos da cultura diversificada do nosso país.

### Contribuições para as áreas da enfermagem, saúde ou políticas públicas

O instrumento validado pode contribuir para a avaliação da percepção das famílias sobre sua capacidade para manejar o cuidado da criança em DP, auxiliando os profissionais de saúde na proposição de ações que favoreçam a autoeficácia da família no cuidado à criança e adolescente em DP no domicílio.

## CONCLUSÕES

A Escala de Percepção da Autoeficácia da Família no Cuidado da Criança com Doença Renal Crônica em Diálise Peritoneal no Domicílio (PAFam-CDR-DP) foi construída para mensurar o quanto a família se percebe capaz para cuidar da criança em DP no domicílio. A validação de conteúdo e a análise semântica indicaram que a escala é adequada e compreensível às famílias que vivenciam esse contexto, e engloba as dificuldades, os desafios e os impedimentos que precisam ser superados por elas.

Recomenda-se dar continuidade ao estudo cumprindo o polo analítico, visando à realização de testes psicométricos para a utilização da escala na prática clínica do enfermeiro com a família nesse contexto.
